# Snakebite Induced Thrombotic Microangiopathy Leading to Renal Cortical Necrosis

**DOI:** 10.1155/2017/1348749

**Published:** 2017-08-13

**Authors:** Ying Mao Gn, Arvind Ponnusamy, Vikram Thimma

**Affiliations:** ^1^Renal Department, Royal Preston Hospital, Preston, UK; ^2^Renal Department, Velammal Medical College Hospital, Madurai, India

## Abstract

Renal complications from snakebite result in high mortality and morbidity. Acute kidney injury (AKI) occurs in 5–30% of cases. Renal manifestation could include acute tubular necrosis, cortical necrosis, interstitial nephritis, glomerulonephritis, and vasculitis. We present a case of thrombotic microangiopathy (TMA) resulting in renal cortical necrosis. Renal biopsy showed fibrin thrombi in glomeruli and arterioles with cortical necrosis. Our patient progressed to end-stage renal disease.

## 1. Introduction

A 60-year-old lady was admitted to our centre 48 hours after an unknown snakebite, having been treated in a peripheral hospital with antisnake venom. There was no relevant previous medical history.

She was intubated on admission with a background of ongoing mucocutaneous bleeding and epistaxis. Endotracheal aspirate was bloody and melena was noted. Her urine output was negligible with frank haematuria. There was an area of cellulitis at the site of snakebite on the left foot. She was hypertensive with BP of 180/70, accompanied with peripheral and pulmonary oedema.

Haematological findings include haemoglobin of 7.1 g/dl, platelet of 33000/cu mm, WCC of 10300/cu mm, bilirubin of 3.47 mg/dl, direct bilirubin of 1.57 mg/dl, SGOT 676 IU/L, SGPT of 165 IU/L, and ALB of 2.5 g/dl with normal clotting. Creatinine level was 4.9 mg/dl with metabolic acidosis. She was transfused with packed red blood cells, fresh frozen plasma, and platelets. Steroids were started under the impression of an acute interstitial nephritis and she was commenced on haemodialysis.

Ultrasound showed a right kidney of 10.3 cm, left kidney of 9 cm, bilateral pleural effusion, dilated IVC, and hepatic veins associated with ascites.

**Figure 1:**
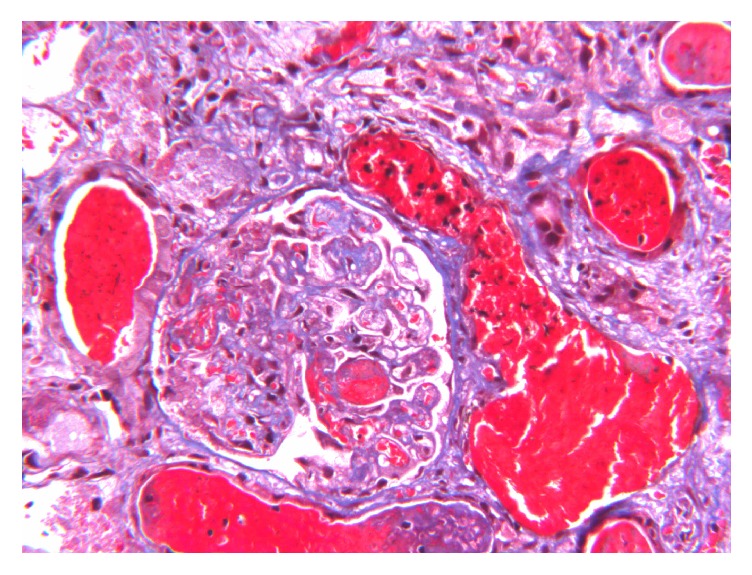
Arterial and arteriolar wall necrosis with luminal occlusion (thrombotic microangiopathy).

**Figure 2:**
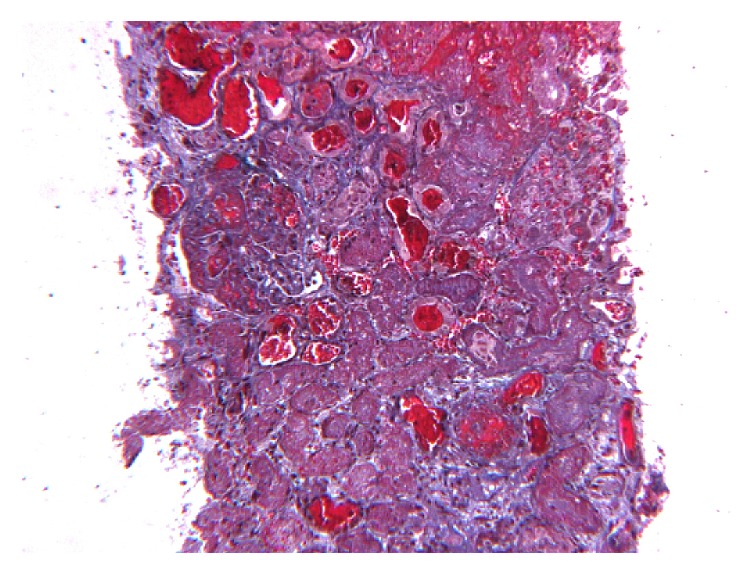
Necrotic process involving 60–70% of sampled cortex.

Renal biopsy revealed renal cortical necrosis with segmental necrosis and luminal thrombotic occlusion in the arteries and arterioles (Figures 1 and 2). Further blood tests revealed an unresolved thrombocytopenia and a blood film showing fragmented red blood cells, suggesting microangiopathic haemolytic anaemia (MAHA). The clinical presentation was therefore consistent with thrombotic microangiopathy (TMA).

LFT normalised on day 5 and haematological parameters including platelets normalised on day 8 with blood pressure control. Renal function did not improve, requiring long-term maintenance haemodialysis, and she was discharged with an arteriovenous fistula.

## 2. Discussion

TMA is characterised by the triad of acute renal failure, thrombocytopenia, and MAHA. At present, there is paucity of literature on TMA following snakebites in contrast to venom-induced consumptive coagulopathy (VICC), a commoner and well-known haematological complication of snakebites [1–3]. This is probably because TMA has been recognised as a complication of snakebites [4] and has been reported in this series of cases [5].

### 2.1. TMA in Snakebites

VICC is the commonest coagulopathy following snake envenomation. It arises from the activation of the coagulation cascade snake toxins including thrombin-like enzymes (most commonly), prothrombin, and factor X activators, resulting in a consumptive coagulopathy [2, 6, 7]. This results in an elevated D-dimer, prolonged prothrombin time, and low fibrinogen, all of which are features that overlap with DIC. However, the activation of the coagulation cascade via a snake procoagulant toxin rather than factor VIIa, the rapid onset and resolution of coagulopathy within 24–48 hours, and the absence of nonrenal end-organ damage seen in VICC make it a separate entity from DIC [1].

TMA tends to occur in conjunction with VICC. However, while the coagulopathy in VICC resolves rapidly, the triad of TMA persists for a longer period of time as seen in the case of our report. It is therefore suggested that the pathological process driving TMA may be distinct but related to that in VICC, although the exact mechanism of TMA in snakebites remains largely unknown. It has been suggested that, in all cases of VICC, there is a potential for TMA to develop but it only manifests in some patients [1]. However, cases of TMA have recently been reported to occur even in the absence of VICC, raising the possibility of an acquired HUS-like syndrome that is independent of VICC [3, 4]. This has led to the suggestion that a toxin in the venom may be precipitating endothelial damage that culminates to TMA [3, 7]. Future research is needed to ascertain the pathophysiology of TMA in snakebites and the relationship between the two conditions.

### 2.2. TMA and Renal Cortical Necrosis

Acute renal failure is a frequently reported complication of snakebites. The pathogenic mechanisms include circulatory collapse following massive haemorrhage, intravascular haemolysis, and VICC [7]. However, most of these cases are self-limiting and resolve completely within 1–8 weeks [1]. Acute tubular necrosis accounts for the large majority of acute renal failure following snakebites [7, 8]. Chronic renal impairment and mortality following TMA in snakebite coagulopathy are uncommon [1]. In this report, however, we observed a case of renal cortical necrosis (RCN) secondary to TMA following a snakebite, an uncommon and severe cause of acute renal failure, resulting in loss of kidney function and end-stage renal disease.

RCN is characterised histologically by ischaemic necrosis of large portions of the renal cortices which is irreversible [9, 10]. Declining urine output is the most common clinical feature described in the literature. The clinical suspicion of RCN should be raised in the context of ARF when oliguria or absolute anuria persists for more than 28 days [8, 10].

RCN is an uncommon cause of renal failure and is usually associated with obstetric complications [10, 11]. It has occasionally been implicated in snakebites [8, 9, 12–14]. However, the current literature available on RCN complicated by TMA is scant. At present, there are scattered reports of a HUS-like syndrome following snakebites, although they have not been recognised as TMA [1]. This could be due to TMA only being recognised recently as an entity on its own which is distinct from VICC. Moreover, since both conditions usually occur in conjunction, with VICC typically dominating the clinical presentation (i.e., haemorrhagic state in our case), a TMA-like syndrome can easily be missed. Furthermore, an isolated thrombocytopenic state in the absence of MAHA can also occur following snakebites [7], making it insufficient for a diagnosis of TMA.

Because of this wide variation in coagulation abnormalities following a snakebite, it would also be interesting to see if there is a correlation between the type and extent of haematological abnormalities and the histological findings in the kidneys.

### 2.3. Clinical Significance

Since the clinical features of TMA and VICC can be similar, prompt investigations should be carried out at the first instance. This includes coagulation studies and a blood film to identify the presence of MAHA. Abnormal results should indicate continuous close monitoring until normalisation. This would allow the early identification of a possible underlying TMA from VICC and guide the choice of treatment. The value of an early renal biopsy remains unknown. Plasmapheresis has been used in cases of TMA following snakebite [5] but there is no evidence to suggest its benefit. In our case, given the lack of clear mechanism for TMA, presence of extensive renal cortical necrosis, oliguria, and dialysis dependence, along with no evidence of benefit, plasmapheresis was not attempted.

Nevertheless, timely administration of antivenom is of paramount importance [15, 16]. Antivenoms are widely used in an attempt to reverse the coagulopathy associated with VICC. However, they cannot reverse any injury or organ damage that has already been caused by the coagulopathy [16]. There is therefore an emphasis on the prompt delivery of antivenoms to limit the deleterious effects of the venom toxin. Interestingly, Isbister et al. also observed that patients who developed MAHA following snakebites tended to receive delayed administration of antivenom, although the study was underpowered to confirm this finding [3].

## 3. Conclusion 

In summary, we report a case of TMA resulting in RCN following a snakebite. A better awareness of TMA and its early identification can contribute to a more tailored approach in the management and better prognosis for the patient. We therefore emphasise the importance of early and complete haematological investigations, including a blood film at presentation in cases following a snakebite.
